# Correlation analysis of MRD positivity in patients with completely resected stage I-IIIA non-small cell lung cancer: a cohort study

**DOI:** 10.3389/fonc.2023.1222716

**Published:** 2023-07-21

**Authors:** Daling Dong, Shixin Zhang, Bin Jiang, Wei Wei, Chao Wang, Qian Yang, Tingzhi Yan, Min Chen, Liken Zheng, Weikang Shao, Gang Xiong

**Affiliations:** ^1^ Department of Cardiothoracic Surgery, Guiqian International Hospital, Guiyang, China; ^2^ Genecast Biotechnology Co., Ltd., Wuxi, China

**Keywords:** molecular residual disease, liquid biopsy, non-small cell lung cancer, adjuvant therapy, clinicopathological features, immune markers

## Abstract

**Background:**

The primary objective of this study is to thoroughly investigate the intricate correlation between postoperative molecular residual disease (MRD) status in individuals diagnosed with stage I-IIIA non-small cell lung cancer (NSCLC) and clinicopathological features, gene mutations, the tumour immune microenvironment and treatment effects.

**Methods:**

The retrospective collection and analysis were carried out on the clinical data of ninety individuals diagnosed with stage I-IIIA NSCLC who underwent radical resection of lung cancer at our medical facility between January 2021 and March 2022. The comprehensive investigation encompassed an evaluation of multiple aspects including the MRD status, demographic information, clinicopathological characteristics, results from genetic testing, the tumor immune microenvironment, and treatment effects.

**Results:**

No significant associations were observed between postoperative MRD status and variables such as gender, age, smoking history, pathological type, and gene mutations. However, a statistically significant correlation was found between MRD positivity and T (tumor diameter > 3 cm) as well as N (lymph node metastasis) stages (p values of 0.004 and 0.003, respectively). It was observed that higher proportions of micropapillary and solid pathological subtypes within lung adenocarcinoma were associated with increased rates of MRD-positivity after surgery (p = 0.007;0.005). MRD positivity demonstrated a correlation with the presence of vascular invasion (p = 0.0002). For the expression of programmed cell death ligand 1 (PD-L1), tumour positive score (TPS) ≥ 1% and combined positive score (CPS) ≥ 5 were correlated with postoperative MRD status (p value distribution was 0.0391 and 0.0153). In terms of ctDNA elimination, among patients identified as having postoperative MRD and lacking gene mutations, postoperative adjuvant targeted therapy demonstrated superiority over chemotherapy (p = 0.027).

**Conclusion:**

Postoperative ctDNA-MRD status in NSCLC patients exhibits correlations with the size of the primary tumor, lymph node metastasis, pathological subtype of lung adenocarcinoma, presence of vascular invasion, as well as TPS and CPS values for PD-L1 expression; in postoperative patients with MRD, the effectiveness of adjuvant EGFR-TKI targeted therapy exceeds that of chemotherapy, as evidenced by the elimination of ctDNA.

## 1 Introduction

In China, Lung cancer holds the unenviable distinction of being the most prevalent malignancy and remains the primary contributor to cancer-related mortality. This devastating disease accounts for a staggering 40% of global deaths attributed to lung cancer, further emphasizing its significant impact on public health. Non-small cell lung cancer (NSCLC) comprises roughly85% of lung cancers, with its predominant pathological subtypes being lung adenocarcinoma and lung squamous cell carcinoma (1, 2). Surgery is one of the main treatments for patients with stage I-IIIA NSCLC. For patients with stage IA and low-risk stage IB NSCLC, regular follow-up and imaging examinations are recommended as the main means of tumour monitoring (3). Adjuvant therapy, encompassing various modalities such as chemotherapy, targeted therapy, immunotherapy, and more, plays a crucial role in the management of postoperative patients with high-risk stage IB and II-IIIA NSCLC. It is crucial to ensure regular clinical follow-ups and imaging assessments during and after the treatment process (4). Despite standardized treatment, there are still patients with stage I-IIIA NSCLC who encounter tumor relapse following surgical intervention (5). Traditional imaging examinations such as computed tomography (CT), as the main means of postoperative tumour monitoring in NSCLC, have certain limitations, such as difficulty in detecting residual or recurring small tumour lesions, and lag behind in the evaluation of chemotherapy efficacy and drug resistance. Detecting small tumour lesions early after radical surgery for NSCLC, prognosticating the likelihood of disease recurrence, and guiding postoperative refined and individualized treatment are the main challenges in the postoperative management of NSCLC.

Circulating tumour DNA (ctDNA) pertains to fragments of tumor-derived DNA present in the bloodstream. These fragments encompass both the phenotype and genetic characteristics of tumour cells, such as gene mutation information, including mutations, deletions, insertions, and rearrangements (6, 7). In recent years, from the perspective of evaluating tumour burden, ctDNA has been used in NSCLC research to evaluate minimal residual disease (MRD). A study of stage I-IIIA NSCLC found that MRD detection based on postoperative plasma ctDNA identify tumour recurrence and metastasis earlier than can imaging. In addition, postoperative ctDNA-MRD-positive patients have a worse prognosis than ctDNA-MRD-negative patients. In addition, it is possible to dynamically monitor postoperative MRD and formulate personalized, precise treatment and/or follow-up plans based on MRD status and abundance changes (8–13). The above studies suggest that MRD detection based on plasma ctDNA serves as a valuable and informative reference for guiding the postoperative management of NSCLC patients and has broad application prospects. However, in the realm of lung cancer, MRD research is still in its nascent stages. Significant heterogeneity exists among studies regarding the definitions and methodologies employed to investigate MRD., and the findings cannot be fully generalized; therefore, relevant research is currently at the preliminary exploration stage of “crossing the river by feeling the stones”.

At the Forum on the 18th China Lung Cancer Summit in 2021, an expert consensus was reached on lung cancer MRD: Lung cancer MRD refers to residual cancer that traditional imaging (including positron emission tomography/CT, PET/CT) or laboratory methods cannot detect after treatment. However, consistent detection of MRD can be achieved through the utilization of liquid biopsy techniques *via* ctDNA (abundance ≥ 0.02%), including lung cancer driver genes or other class I/II gene variants, representing the possibility of lung cancer persistence and clinical progression (14). In a recent extensive prospective study conducted across multiple medical centers, it was conclusively shown that ctDNA can serve as a reliable biomarker for prognosticating and detecting MRD within a span of one month following surgical intervention, and the relative contribution of ctDNA-MRD status in multivariate Cox analysis in predicting recurrence-free survival (RFS) surpasses the cumulative impact of clinical variables, including s TNM stage (15). However, there is no detailed literature report on whether the MRD status of NSCLC patients after radical surgery is related to clinicopathological characteristics, gene mutations, tumour immune biomarkers and other factors.

In this study, we retrospectively conducted a comprehensive analysis of a cohort comprising 90 individuals diagnosed with stage I-IIIA NSCLC who had undergone radical surgery in our hospital. The peripheral blood of the patients was analysed by next-generation sequencing (NGS) technology covering 769 cancer-related genes, and the correlation between ctDNA-MRD status and clinicopathological features, gene mutations, the tumour immune microenvironment and treatment efficacy was analysed. By examining these variables collectively, we aimed to investigate the relationship among MRD status, clinicopathological characteristics and treatment prognosis in NSCLC patients.

## 2 Methods

### 2.1 Patients and samples

Patients with stage I-IIIA disease (AJCC8th edition) who underwent radical surgery (lobectomy + systematic lymph node dissection) for NSCLC from January 2021 to March 2022 at the Department of Thoracic and Cardiovascular Surgery of Guiqian International General Hospital and agreed to undergo MRD testing were included. Patients with a prior history of neoplastic disease, neoadjuvant therapy, multiple primary lung cancers, compound carcinoma, and germline mutations were excluded. For eligible patients, the excised pathological tissue was fixed in formalin solution and subsequently embedded in paraffin for the purpose of sectioning following surgery. Subsequently, the peripheral blood of each patient was collected for MRD detection 1 month after the operation. If the sample was MRD positive, adjuvant therapy was administered considering the gene test results, and the peripheral blood of the patient was collected again 3 months after the end of the adjuvant treatment for MRD detection. If the patient’s peripheral blood MRD test was negative 1 month after surgery, adjuvant therapy was administered based on the stage, and peripheral blood was collected 6 months after surgery for MRD detection regardless of whether the patient received adjuvant therapy. The requirements for pathological tissue sections of patients were as follows: tumour positive score (TPS) ≥10%, area of necrotic tissue ≤50%, and section thickness of 5-10 μm. Streck blood tubes were used for peripheral blood collection, and each blood collection volume was at least 10 ml. Tissue and blood samples were sent to Genecast Biotechnology Co., Ltd. (Wuxi,China) for DNA isolation, library preparation, and NGS. Regular follow-up visits were scheduled for all patients at three-month intervals post-operation. The follow-up protocol encompassed a comprehensive evaluation, including a review of medical history, physical examination, chest CT scan, and abdominal B-ultrasound, as well as yearly cranial MRI and whole-body bone scans. Prior approval for this study was obtained from the Ethics Committee of Guiqian International General Hospital, and written informed consent was obtained from all participating patients, ensuring adherence to ethical guidelines and patient autonomy.

### 2.2 DNA extraction from tissue and blood samples

The extraction of genomic DNA from tissue slices was performed using the TIANamp Genomic DNA Kit (TIANGEN, China) following established protocols. After centrifugation, TGuide S32 Magnetic Blood DNA Kit-T5C and TGuide S32 (TIANGEN, China) automatic nucleic acid extractor were employed for extracting genomic DNA from the buffy coat fraction. Similarly, isolation of cell-free DNA (cfDNA) was extracted from the plasma fraction using MagMAX Cell-Free DNA Isolation (ThermoFisher, USA). The quantification of DNA concentration was performed utilizing a Qubit dsDNA HS Assay Kit (Thermo Fisher, USA), and DNA quality was assessed using an Agilent 2100 BioAnalyzer (Agilent, USA). Each tumor tissue or plasma sample yielded a range of 30 to 300 ng of genomic DNA, which was then subjected to shearing using Covaris LE220 to achieve a fragment length of 200 bp. The resulting DNA fragments underwent further processing and were deemed qualified for subsequent library preparation.

### 2.3 Library preparation and sequencing

A KAPA Hyper PCR-free kit was used to construct a DNA library, and the library was amplified using a KAPA Library Amplification Kit and purified using Agencourt AMPure XP magnetic beads. UMI connectors were added to both ends of the DNA. The quantification of modified library samples was carried out using the AccuGreen High Sensitivity dsDNA Quantification Kit (Biotium, USA), enabling accurate measurement of library sample concentration. Additionally, the size distribution of the libraries was evaluated using an Agilent Bioanalyzer 2100 (Agilent, USA).

The HyperCap Target Enrichment Kit (Roche, Switzerland) was employed to capture the desired genomic regions of interest. The designed hybridization panel covered a region of approximately 2.4 MB in the human genome, covering 769 cancer-related genes. The 769-gene NGS panel was developed and validated in-house by Genecast Biotechnology, a CAP-accredited clinical diagnostic laboratory. The genes included in the panels are curated according to publicly available databases, including The Cancer Genome Atlas (TCGA, https://www.cancer.gov ), the Catalogue of Somatic Mutations in Cancer (COSMIC, https://cancer.sanger.ac.uk ), OncoKB (https://www.oncokb.org ), etc., and proprietary internal datasets. Following hybridization and subsequent washing steps as per the provided instructions, the enriched libraries underwent amplification using KAPA HiFi HotStart ReadyMix. The amplified libraries were then purified using 1X AMPure beads, quantified to determine their concentration, and subjected to sequencing in 150-bp paired-end mode using the Illumina NovaSeq 6000 platform.

### 2.4 Identification of single-nucleotide variants in tumour tissue

Trimming of aptamers and low-quality bases for sequencing reads was performed using Trimmomatic (v0.36) (16). The obtained high-quality reads were aligned to the human reference genome hg19 using BWA aligner (v0.7.17), followed by Picard (v2.23.0) for classification and masking of duplicates. To identify SNVs and insertions/deletions (InDels), VarDict (version 1.5.1) analysis was employed in this study. Additionally, FreeBayes (version 1.2.0) was utilized to detect complex mutations (17). A typical quality check (QC) was used to filter the raw variant list, for example, variant quality and chain bias. Additionally, variants classified as low complexity and segmental repeat regions defined by ENCODE were removed (18), and variants on a list of recurring sequence-specific errors (SSEs) were developed and validated in-house.

### 2.5 Filtering of point mutations in tumour tissue

Filtering was performed first if germline or clonal haematopoiesis met any of the following criteria: 1) variant allele frequency (VAF) in peripheral blood lymphocytes (PBLs) not less than 5%; 2) VAF in PBLs less than 5% but greater than 1/5 of the VAF in paired tissue samples; and 3) variants found to have a minimum allele frequency (MAF) no less than 2% in the public gnomAD population database. The remaining somatic mutations were subjected to further quality filtering. The minimum supported reads was 5 (19), and the VAF thresholds were 4% for SNVs and 5% for InDels.

### 2.6 Monitoring point mutations in plasma samples

To ensure the accuracy and reliability of identified SNVs/InDels, each potential ctDNA mutation candidate underwent rigorous statistical testing against an internal background reference library. We adopted a tumor prior analysis strategy for mutational analysis of plasma samples, whereby somatic SNVs/InDels identified in each tissue sample constituted the baseline for ctDNA detection in corresponding plasma cfDNA samples from the same patient; in the initial tissue sequencing, novel ctDNA mutations that were not detected were excluded from the data analysis. In order to mitigate the influence of technical artifacts, each identified ctDNA mutation candidate underwent a rigorous statistical analysis against an internal background reference library. The background library included more than 1,000 plasma samples as well as matched tissue and peripheral blood cell samples from patients of various stages and cancer types. After the removal of true tumour-derived somatic mutations and clonal haematopoietic (CH) variants by reference to matched tissue and PBL sequences, the remaining artificial variants in the background library were merged at the minor allele level and fitted with anti-γ distribution. Each input plasma sample to be analysed first underwent the same mutation filtering process to remove germline and CH variants, with the remaining candidate somatic mutations statistically tested against a background reference library. To account for the zero-inflation effect, the zero weighted probability that a given variant is a true somatic cell was calculated, by Monte Carlo simulations, combining randomly sampled nonzero VAF values with all zero values. Each VAF in this list was further entered into a binomial test as a probability of success with the parameters observed alt-allele support reads and total support reads. Expected values at the variant level were calculated as the mean of all P values, and a threshold for positive ctDNA mutations was set at P < 0.05. The following formula was used to calculate the comprehensive P value at the sample level: Psma pte = C^km^nPi , where m of the combination coefficient (C) is the total number of variants tracked for this patient and k is the number of positive variants tested by the variant level above. A plasma sample was designated as positive using an integrated P value threshold of P < 0.01. The mean VAF for a given positive plasma sample was calculated by dividing the sum of the VAF values of all positive SNV/indel variants by the number of all traceable variants. The haploid genome equivalent (hGE, mutant molecules/ml plasma) used to assess ctDNA concentration was calculated using the following formula:

mean V AF *cfDN A concertration ( ng jml pl amsa }

o.ooa; *(ng /genome )*

### 2.7 Identification and monitoring of copy number variation in plasma samples

Copy number variation (CNV) for both tissue and plasma samples were analysed using CNV kit (v0.9.2) software based on paired PBL samples (20). For tissue, a copy number threshold of 4.0 was applied to identify CNV gains, while a threshold of 1.0 was used for CNV losses in the tissue samples. In contrast, for plasma samples, the copy number thresholds were set at 3.0 for CNV gains and 1.2 for CNV losses. In the case of plasma samples, a positive report for CNVs was only assigned if the corresponding gene-level copy number alteration (either an increase or decrease) was also identified in the initial resected baseline tissue sample.

### 2.8 Detection and monitoring of gene fusions in plasma samples

Gene fusions were identified with FACTERA v1.4.4 and FusionMap (21, 22), and only typical driver fusions involving kinase domains that activate ALK, ROS1, and RET were included in the analysis. Clean plasma cfDNA sequences were mapped to fusion references from corresponding tissue samples. Plasma samples were defined as fusion positive if at least 1 sequencing read exactly matched the reference and spanned the breakpoint.

### 2.9 Tumour marker-related detection

PD-L1: PD-L1 expression was assessed utilizing a Dako 22C3 kit, a well-established method for analyzing PD-L1 protein levels. The scoring system employed in this study was based on the tumor proportion score (TPS), which represents the percentage of PD-L1 membrane-positive tumor cells within the total number of tumor cells evaluated. (TPS ≥ 1% is positive, TPS < 1% is negative). The combined positive score (CPS) is a metric used to evaluate PD-L1 expression levels. It is calculated by dividing the total number of PD-L1-positive cells (including tumor cells, lymphoid cells, and macrophages) by the total number of visible tumor cells assessed, and then multiplying the result by 100 (CPS ≥ 5 is positive, CPS < 5 is negative).

Tumour mutational burden (TMB): TMB (nonsynonymous mutations per megabase (Mbase) of DNA) was calculated using sequencing data from a panel of 769 cancer-related genes and determined by assessing the number of nonsynonymous somatic mutations per megabase (Mb) of the genome. In this study, quantiles < 25% were considered TMB-L, quantiles ≥ 25% and < 75% were considered TMB-M, and quantiles ≥ 5% were considered TMB-H.

Human leukocyte antigen (HLA): Trimmomatic (V0.39) was used to trim the adaptor of raw read pairs. The obtained high-quality reads were aligned to the human reference genome (HG19, UCSC assembly) using the BWA-MEM aligner (version 0.7.12). Subsequently, the alignment files were processed and analyzed using SAMtools (version 1.3). GATK V2.8 was used to perform markduplicates, and local INDEL rearrangement was performed. The HLA gene region in the markduplicated BAM file was converted to FASTQ format and input into HLA-HD (V1.2.0.1) for HLA allele type analysis (MINMUM_TAG_SIZE, 50; RATE_OF_CHUTING, 0.95). The method involved building an extensive dictionary of HLA alleles. Finally, the HLA allele types were analysed.

### 2.10 Adjuvant therapy

The adenocarcinoma chemotherapy regimen (AP regimen) was as follows: pemetrexed + platinum (cisplatin/carboplatin); specific dosage: pemetrexed 500 mg/m2 q3w ivgtt, carboplatin 200-400 mg/m2 q3w ivgtt, and cisplatin platinum 50~100 mg/m2 q3w ivgtt, 4-6 cycles. The chemotherapy regimen for squamous cell carcinoma (TP regimen) was a follows: nab-paclitaxel + platinum (cisplatin/carboplatin); specific dosage: nab-paclitaxel 260 mg/m2 q3w ivgtt, carboplatin 200-400 mg/m2 q3w ivgtt, and cisplatin 50~100 mg/m2 q3w ivgtt, 4-6 cycles. Tislelizumab (200 mg q3w ivgtt) was used as the immune checkpoint inhibitor. The choice of targeted therapy was guided by the findings of genetic testing. Based on the specific genetic alterations identified, appropriate targeted therapies were selected. In this study, gefitinib (250 mg once daily, orally) was administered as a first-generation EGFR-TKI, while osimertinib (80 mg once daily, orally) was utilized as a third-generation EGFR-TKI.

### 2.11 Statistical analyses

The mutation status of lung cancer patients was visualized using ComplexHeatmap, and statistical analysis was performed using RStudio software (version 4.1.2; RStudio Inc). Independent samples t-tests were employed to compare continuous variables, while chi-square tests were utilized to assess differences in categorical variables. In both cases, a significance level of P < 0.05 was used as the threshold to determine statistical significance.

## 3 Results

### 3.1 Patient characteristics

**Figure 1:**
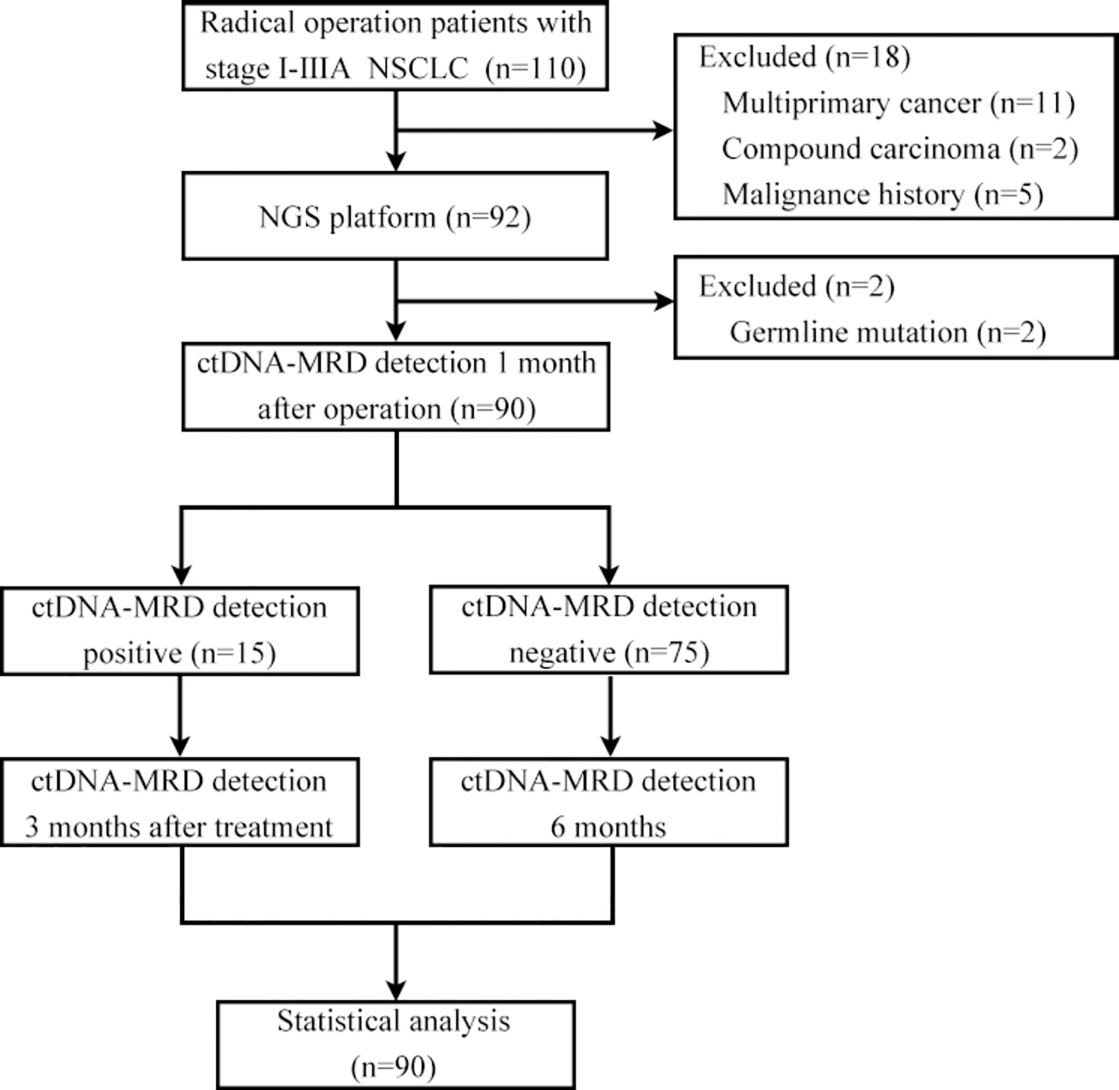
Flow chart of patient enrolment.

A cohort comprising 110 patients with stage I-IIIA NSCLC underwent radical surgery and agreed to undergo postoperative ctDNA-MRD detection. Eleven patients with multiple primary lung cancer, 2 patients with compound carcinoma, 5 patients with previous malignant tumour, and 2 patients with germline inheritance were excluded. A total of 90 NSCLC patients met the inclusion criteria for the analysis, of whom 15 (16.67%) were ctDNA-MRD positive and 75 (83.33%) were ctDNA-MRD negative. The median duration of postoperative follow-up was 240 days, with a range from 120 to 360 days. During this period, no radiographic evidence of recurrence was detected in any of the patients included in the study (**Figure 1**).

Among the 90 patients with NSCLC, the median age was 55 years (ranging from 35 to 75 years). Out of these, there were 39 male patients (43.33%) and 51 female patients (56.67%). There were 78 patients (86.67%) with lung adenocarcinoma and 12 patients (13.33%) with lung squamous cell carcinoma. Postoperative pathological staging revealed 41 patients (45.56%) with stage IA disease, 5 patients (5.56%) with stage IB disease, 1 patient (1.11%) with stage IIA disease, 12 patients (13.33%) with stage IIB disease, and 31 patients (34.44%) with stage IIIA disease. All 12 patients with squamous cell carcinoma received adjuvant therapy (4 patients received the TP regimen, and 8 patients received TP + tislelizumab). Thirty-two patients with adenocarcinoma received adjuvant therapy (20 patients received the AP regimen, 4 patients received oral gefitinib, and 8 patients received oral osimertinib (**Supplementary Table S1**).

### 3.2 Correlation between clinicopathological features and postoperative MRD status

MRD-positive patients had a median age of 52 years (range: 46-55 years), whereas MRD-negative patients had a median age of 55 years (range: 35-75 years). There was no statistical significance between age and postoperative MRD status (t = 1.5364, df = 33.012, p value = 0.1341) (**Table 1**). Among the MRD-positive patients, 7 (7/15, 46.67%) were female, and 8 (8/15, 53.33%) were male; among the MRD-negative patients, 44 (44/75, 58.67%) were female, and 31 (31/75, 41.33%) were male. There was no significant difference between sex and postoperative MRD status (X-squared = 0.70769, df = 1, p value = 0.4002) (**Table 1**). Five MRD-positive patients (5/15, 33.33%) had a smoking history [(smoking index (660 ± 150)], and 13 MRD-negative patients (13/75, 17.33%) had a smoking history [smoking index (940 ± 860)]. The smoking index was not significantly associated with MRD positivity (X-squared = 0.05395, df = 1, p value = 0.8163) (**Table 1**).

**Table 1:** Patient clinical characteristics.

Characteristic	MRD positive	MRD negative	*p* value
Age (years)	52 (46-60)	55 (35-75)	0.1341
Sex			0.4002
Female	7	44	
Male	8	31	
Smoking history			0.8163
YES	5	13	
Smoking index	660 ± 150	940 ± 860	
NO	10	62	
Pathological type			0.6926
LUAD	11	67	
LUSC	4	8	
Pathological subtype (LUAD)			0.007
LPA-based	0	16	
ACI-based	2	39	
PAP-based	0	6	
MPA-based	3	1	
SPA-based	6	5	
Differentiation (LUSC)			0.005
Well Moderate	0 2	1 6	
Poorly	2	2	
TNM staging			0.004
Stage IA	0	41	
Stage IB	0	5	
Stage IIA	0	1	
Stage IIB	3	9	
Stage IIIA	12	19	
Tumour size			0.0040
≤3 cm	4	57	
>3 cm	11	18	
Lymph node metastasis			
LUAD			0.0030
YES	11	16	
NO	0	51	
LUSC			0.0004
YES	4	1	
NO	0	7	
Vascular invasion			0.0002
YES	13	23	
N0	2	52	
Nerve invasion			0.3636
YES	6	11	
N0	9	64	

LUAD, lung adenocarcinoma; LUSC, lung squamous cells; LPA, lepidic predominant adenocarcinoma; ACI, acinar predominant adenocarcinoma; PAP, papillary predominant adenocarcinoma; MPA, micropapillary predominant adenocarcinoma.

Among the 78 patients with lung adenocarcinoma, 11 (14.10%) had a positive MRD status after the operation, and 67 (85.90%) had a negative MRD status after the operation. Among the 12 patients with squamous cell lung carcinoma, 4 (33.33%) had a positive postoperative MRD status, and 8 (66.67%) had a negative postoperative MRD status. There was no statistical significance between pathological type and postoperative MRD status (X-squared = 0.15624, df = 1, p value = 0.6926) (**Table 1**).

Among the pathological subtypes of lung adenocarcinoma, there were 16 cases of lepidic predominant adenocarcinoma (LPA) with a negative postoperative MRD status; 41 cases of acinar predominant adenocarcinoma (ACI), including 2 patients (4.88%) with a positive MRD status after surgery and 39 patients (95.12%) with a negative MRD status after surgery; 6 cases of papillary predominant adenocarcinoma (PAP) with a negative postoperative MRD status. There were 4 cases of micropapillary predominant adenocarcinoma (MPA), including 3 patients (75.00%) with a positive postoperative MRD status and 1 patient (25.00%) with a negative postoperative MRD status. There were 11 cases of solid predominant adenocarcinoma with mucin production (SPA), including 6 patients (54.55%) with a positive postoperative MRD status and 5 patients (45.45%) with a negative MRD status. Lung adenocarcinoma patients with higher proportions of SPA and MPA components had a higher rate of postoperative positive MRD status (X-squared = 27.088, df = 5, p value = 0.005) (**Table 1**). Among the patients with squamous cell carcinoma, 1 case was well differentiated, and the postoperative MRD status of the patient was negative; 8 cases were moderately differentiated, including 2 patients (25.00%) with a positive postoperative MRD status and 6 patients (75.00%) with a negative postoperative MRD status; and 3 cases were poorly differentiated, including 1 patient (33.33%) with a positive MRD status after surgery and 2 patients (66.67%) with a negative MRD status after surgery. There was no significant difference in positive MRD status after surgery among squamous cell carcinoma patients with different tumour differentiation (X-squared = 0.4444, df = 2, p value = 0.800) (**Table 1**).

We stratified the sample by largest diameter of the lung tumour exceeding 3 cm. There were 61 patients with T ≤ 3 cm, of whom 4 patients (6.56%) had a positive postoperative MRD status and 57 patients (93.44%) had a negative postoperative MRD status. There were 29 patients with T > 3 cm, of whom 11 (37.93%) had a positive postoperative MRD status and 18 (62.07%) had a negative postoperative MRD status. T > 3 cm was positively correlated with positive postoperative MRD status (X-squared = 18.153, df = 3, p value = 0.0004) **Table 1**).

Among the 78 patients with lung adenocarcinoma, 27 had lymph node metastasis, of whom 11 (40.74%) had a positive postoperative MRD status and 16 (59.26%) had a negative postoperative MRD status. Fifty-one patients had no lymph node metastasis, and their postoperative MRD status was negative. Lymph node metastasis was associated with postoperative MRD positivity in lung adenocarcinoma patients (X-squared = 18.153, df = 3, p value = 0.003) (**Table 1**). Among the 12 patients with squamous cell lung carcinoma, 5 had lymph node metastasis, among whom 4 patients (80.00%) had a positive postoperative MRD status and 1 (20.00%) had a negative postoperative MRD status. Seven patients had no lymph node metastasis, and their postoperative MRD status was negative. Positive postoperative MRD status in patients with squamous cell lung carcinoma was found to be associated with lymph node metastasis (X-squared = 18.153, df = 3, p value = 0.0004) (**Table 1**).

Of the 36 patients with vascular invasion, 13 (36.11%) had a positive postoperative MRD status, and 23 (63.89%) had a negative postoperative MRD status. Of the 54 patients without vascular invasion, 2 (3.70%) had a positive postoperative MRD status, and 52 (96.30%) had a negative postoperative MRD status. Vascular invasion was associated with positive postoperative MRD status (X-squared = 13.587, df = 1, p value = 0.0002) (**Table 1**). Of the 17 patients with nerve invasion, 6 (35.30%) had a positive postoperative MRD status, and 11 (64.70%) had a negative postoperative MRD status. Of the 73 patients without nerve invasion, 9 (12.33%) had a positive postoperative MRD status, and 64 (87.67%) had a negative postoperative MRD status. There was no statistical correlation between nerve invasion and postoperative MRD-positive status (X-squared = 4.3802, df = 1, p value = 0.3636) (**Table 1**).

### 3.3 Gene mutations associated with postoperative MRD status

**Figure 2:**
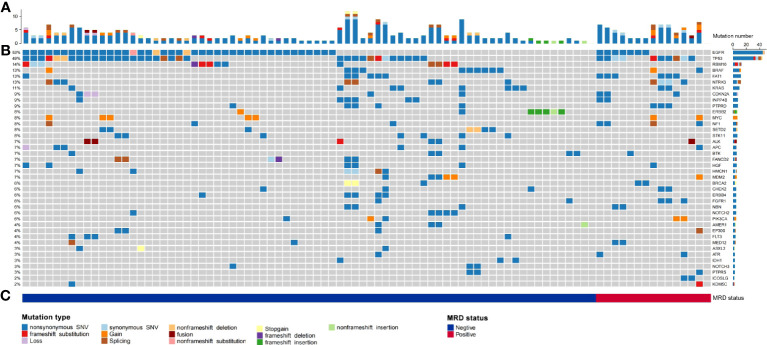
Summary of the number of physical mutated genes in tube issue, mutation type, and periodic ctDNA testing in 90 evaluable patients with NSCLC. **(A)** Number of physical mutations in tube issue. **(B)** Candidate driver genes that are frequently mutated in tube issue. **(C)** Patient's mutation type, and MRD status.

Gene mutations detected by NGS in tumour tissue were mainly point mutations, accounting for 88.41% (203 point mutations); fusions and rearrangements accounted for only 11.59% (26 fusions and rearrangements) (**Figure 2A**). The most frequently mutated genes were EGFR (53%), TP53 (49%), BRAF (13%), NTRK3 (13%), KRAS (11%), ERBB2 (8%), and ALK (7%) (**Figure 2B**). EGFR, TP53, BRAF, NTRK3, KRAS, ERBB2, and ALK gene mutations were not significantly associated with MRD-positive status (**Figures 2B, C**; **Supplementary Table S2**).

### 3.4 Correlation between immune biomarkers and MRD status

**Figure 3:**
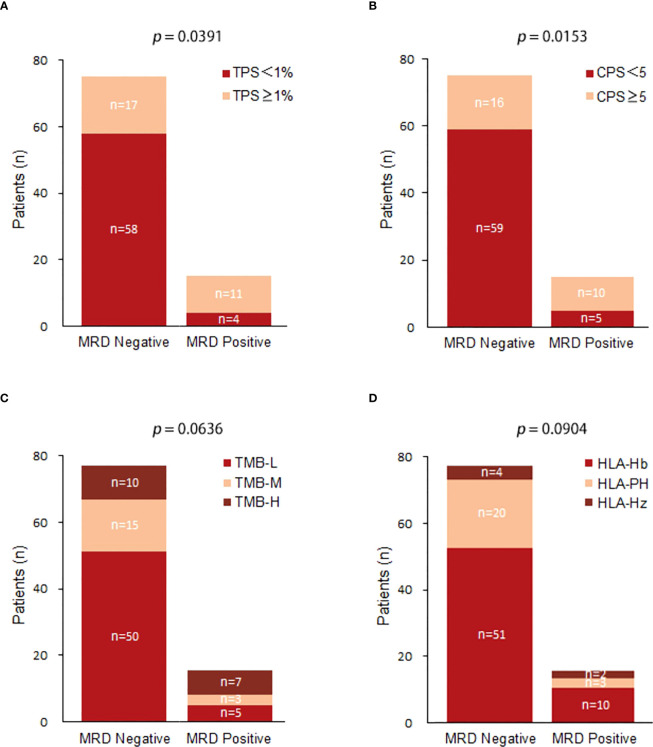
**(A)** Comparison of TPS(Tumor Positive Score) expression between postoperative MRD-positive patients and MRD-negative patients; **(B)** Comparison of CPS(Combined Positive Score ) expression between postoperative MRD-positive patients and MRD-negative patients; **(C)** Comparison of TMB expression between postoperative MRD-positive patients and MRD-negative patients.(TMB-L:Tumor Mutational Burden-Low;TMB-M:Tumor Mutational Burden-middle;TMB-H:Tumor Mutational Burden-High); **(D)** Comparison of HLA between postoperative MRD-positive patients and MRD-negative patients.(HLA-Hb : Human leukocyte antigen-;HLA-PH;HLA-HZ).

Among MRD-positive patients, 4 patients (26.67%) had a TPS < 1%, and 11 patients (73.33%) had a TPS ≥ 1%; among MRD-negative patients, 58 patients (77.33%) had a TPS < 1%, and 17 patients (22.67%) had a TPS ≥ 1%. TPS ≥ 1% was significantly associated with postoperative MRD-positive status (X-squared = 4.2558, df = 1, p value = 0.0391) (**Figure 3A**). Among MRD-positive patients, 5 patients (33.33%) had a CPS < 5, and 10 patients (66.67%) had a CPS ≥ 5; among MRD-negative patients, 59 patients (78.67%) had a CPS < 5, and 16 patients had a CPS ≥ 5. (21.33%). CPS ≥ 5 was significantly associated with postoperative MRD-positive status (X-squared = 0.53321, df = 1, p value = 0.0153) (**Figure 3B**). Among MRD-positive patients, there were 5 (33.33%) TMB-L patients, 3 (20.00%) TMB-M patients, and 7 TMB-H patients (46.67%); among MRD-negative patients, there were 50 TMB-L patients (66.67%), 15 (20.00%) TMB-M patients, and 10 TMB-H patients (13.33%). No substantial association was observed between TMB and MRD status.(X-squared = 1.8646, df = 2, p value = 0.0636) (**Figure 3C**). Among MRD-positive patients, 2 (13.33%) were HLA homozygous, 3 (20.00%) were partially HLA homozygous, and 10 (66.67%) were HLA heterozygous; among MRD-negative patients, 4 were HLA homozygous (5.33%), 20 (26.67%) were partially HLA homozygous, and 51 (68.00%) were HLA heterozygous. No significant relationship was identified between HLA expression and postoperative MRD status (X-squared = 4.8062, df = 2, p value = 0.0904) (**Figure 3D**).

### 3.5 MRD status after adjuvant therapy

**Figure 4:**
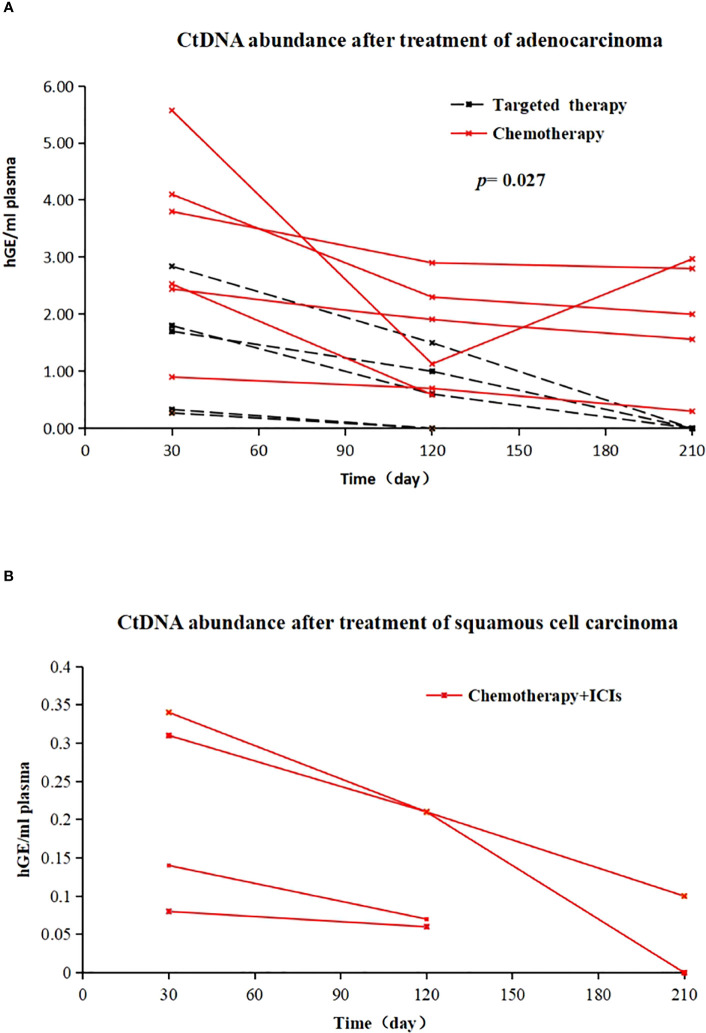
**(A)** Changes in ctDNA abundance after EGFR-TKI treatment (dotted line) and chemotherapy (solid line) in 11 postoperative MRD-positive adenocarcinoma patients; X-axis represents follow-up time, Y-axis represents ctDNA abundance; **(B)** in vivo tumour ctDNA abundance in 4 patients with MRD-positive squamous cell carcinoma treated with chemotherapy + ICIs; X-axis represents follow-up time, Y-axis represents ctDNA abundance.

Of the 11 patients with MRD-positive lung adenocarcinoma, 6 (6/11, 54.55%) were treated with chemotherapy (AP regimen), and 5 (5/11, 45.45%) were treated with EGFR-TKIs (1 with oral gefitinib and 5 cases of oral osimertinib). Among chemotherapy patients, ctDNA abundance decreased after treatment in 5 patients, and ctDNA abundance decreased first and then increased after treatment in 1 patient. The MRD of EGFR-TKI-treated patients became negative (the average treatment time was 126 days). Patients who received postoperative adjuvant EGFR-TKI demonstrated a more effective clearance of ctDNA compared to those who received chemotherapy. (X-squared = 3.2158, df = 1, p value = 0.027) (**Figure 4A**). All 4 patients with MRD-positive squamous cell carcinoma received chemotherapy + immunotherapy (TP regimen + tislelizumab) after surgery. After treatment, 3 patients were still MRD positive, but ctDNA abundance showed a downwards trend; for 1 patient, MRD status became negative after treatment (**Figure 4B**).

## 4 Discussion

With continuous improvements in ctDNA detection technology, postoperative MRD detection has increasingly recognized that liquid biopsy serves as a significant tool for prognostication and assessing treatment response in patients diagnosed with NSCLC (23). For solid tumours, the methods for ctDNA-MRD detection *via* peripheral blood are diverse. Abbosh tracked variations in plasma with tumour-personalized sequencing-focused multidimensional PCR panels. Parikh (24), independent of tumour gene sequencing, established comprehensive genomic and genetic analyses by building a maximized plasma sample bank to identify variant genes in blood. The technical approach by LUNGCA (15) was created on the basis of personalized sequencing, and a negative plasma background reference library was established; each candidate ctDNA mutation was underwent a statistical analysis against an internal reference library. This rigorous assessment was performed to distinguish true somatic mutations from potential confounding germline or CH alterations., in combination with the P value method to determine the MRD status of samples, leading to improved accuracy and reliability in identifying genetic alterations. In this study, the same technical method as that in the LUNGCA study was used to detect ctDNA in peripheral blood, and ctDNA abundance ≥ 0.008% was defined as MRD positive, which is higher than the standard ctDNA abundance ≥ 0.02% required by the expert consensus on MRD in lung cancer. False negatives in MRD interpretations were avoided, which ensured the reliability of the research results.

In the analysis of the basic data of patients, 56.67% of the patients were female, 86.7% of the cancer cases were adenocarcinoma, and 51.3% of the patients had stage I disease, findings that are consistent with the baseline data reported in previous MRD studies (25). This indicates that early-stage female adenocarcinoma is predominant among NSCLC patients currently undergoing surgical treatment. Furthermore, the findings from this investigation indicated no significant associations between postoperative MRD status and patient age, sex, smoking index, or pathological type.

Previous studies have revealed that within the pathological subtypes of lung adenocarcinoma, MPA and SPA types are typically associated with a higher incidence of lymph node metastasis and poorer prognosis (26–29). Our study found that among 78 patients with lung adenocarcinoma, 11 patients (14.10%) had a positive postoperative MRD status; among them, 9 patients had the pathological subtypes MPA and SPA, and the other 2 patients had ACI, but each had 35% micropapillary component and 20% solid component, showing that MPA and SPA were positively correlated with MRD positivity. No significant correlation was observed between the degree of differentiation in squamous cell carcinoma patients and their MRD status., a result that may be due to the small number of squamous cell carcinoma cases. We will supplement these data in a follow-up prospective study.

Our study also revealed a positive correlation between a primary lesion’s largest diameter exceeding 3 cm and a postoperative positive MRD status. Previous research has elucidated that in the early stages of lung cancer, nutrients and oxygen are predominantly acquired through diffusion. However, as the tumor volume increases, angiogenesis is induced by the release of vascular endothelial growth factors (VEGFs) to ensure an adequate supply of nutrients (30). The VEGF family includes VEGF-A, VEGF-B, VEGF-C, VEGF-D, VEGF-E, VEGF-F, etc. Among them, VEGF-A plays a pivotal role as a potent regulator of angiogenesis. It exerts control over vascular permeability and facilitates the metastasis of endothelial cells. The close association between VEGF-A and tumor growth and metastasis highlights its significance in tumor progression and dissemination (31). As tumour volume increases, the release of VEGF-A increases, angiogenesis increases, and vascular permeability increases, resulting in an increase in the release of metabolites containing tumour-associated DNA and biologically active micrometastases into the bloodstream (32); these fragments containing tumour genetic material form ctDNA. This is also consistent with the conclusion in this study that tumour size is correlated with postoperative MRD-positive status.

Lymph node metastasis represents an independent prognostic risk factor for patients with lung cancer. The process of lymph node metastasis in lung cancer primarily occurs through two principal mechanisms: the dissemination of tumor cells *via* the lymphatic vessels within the lungs and the induction of lymphangiogenesis by the tumor itself (31, 33). VEGF-C acts as the main driver during lymphangiogenesis, activates the extracellular regulated protein kinases (ERK 1) or ERK2 pathways, plays a significant role in promoting the growth and development of lymphatic endothelial cells (LECs). It facilitates the process of lymphangiogenesis, leading to the formation of new lymphatic vessels surrounding the tumor. Moreover, VEGF-C increases the permeability of peritumoral lymphatic vessels, facilitating nutrient supply and aiding in the growth and progression of the tumor. When multiple lymphatic pathways are established between a tumour and its surroundings, the tumour releases tumour-associated DNA fragments or micrometastases into the blood through lymphatic backflow (34). Furthermore, VEGF-C demonstrates upregulation in numerous cancer cell types and can possess the ability to indirectly trigger infiltration, as well as migration of stromal macrophages in tumours, thereby promoting the metastasis (34, 35). This also explains the positive correlation between lymph node metastasis and postoperative MRD-positive status in this study.

PD-1/PD-L1 plays a crucial role as a coinhibitory signalling pathway that protects cells from autoimmune or inflammatory cell attack in healthy individuals. The correlation between PD-L1 expression and the prognosis of lung cancer patients remains a topic of debate. However, a growing body of research indicates that the PD-1/PD-L1 signaling pathway contributes to oncogene transcription and evasion, ultimately fostering tumor cell survival and proliferation (36). A meta-analysis of 3107 patients with solid tumours by Wu et al. (36) showed that in contrast to patients with negative PD-L1 expression, those exhibiting high PD-L1 expression demonstrated poorer overall survival (OS). Furthermore, the analysis revealed a positive correlation between PD-L1 expression and tumor T stage, with PD-L1 expression being significantly higher in larger-diameter tumors compared to smaller ones. This observation suggests that elevated PD-L1 expression facilitates immune escape by promoting tumor growth and evasion of the immune system. A meta-analysis by Wen et al. (37) included 254 studies with a total of 1819 patients, and the results showed that PD-L1-positive expression was associated with poorer OS and disease-free survival (DFS) in urothelial carcinoma patients. In this study, among patients with minimal residual disease (MRD), a higher proportion of individuals exhibited PD-L1 tumor proportion score (TPS) ≥ 1% and combined positive score (CPS) ≥ 5, indicating that those with elevated PD-L1 expression experienced a more unfavorable postoperative prognosis.

ADAURA (38) also demonstrated that adjuvant targeted therapy, compared with placebo, after NSCLC surgery has the potential to substantially enhance the DFS outcomes in patients. EVAN (39) showed that in patients with stage IIIA NSCLC with R0 resection and a sensitive EGFR gene mutation, postoperative targeted therapy with erlotinib can benefit patients more than can chemotherapy. This study found that in patients with MRD-positive lung adenocarcinoma, targeted therapy was better than chemotherapy alone for residual ctDNA clearance, which partly provides an explanation for the enhanced DFS and improved prognosis observed in patients undergoing targeted therapy.

Our study has limitations. 1) Given the limited sample size and retrospective nature of this study conducted at a single center, it is important to note that the data may be subject to some degree of skewness. 2) The duration of patient follow-up was relatively brief, and the relationship between MRD status and DFS and OS could not be determined. 3) The effectiveness of postoperative adjuvant therapy for MRD-negative patients still requires evaluation in subsequent studies.

## 5 Conclusion

In conclusion, our study revealed a correlation between postoperative ctDNA-MRD status in NSCLC patients and various factors including primary tumor size, lymph node metastasis, lung adenocarcinoma subtype, vascular invasion, as well as TPS and CPS for PD-L1 expression. Additionally, among MRD-positive patients, adjuvant EGFR-TKI targeted therapy demonstrated superior clearance of ctDNA compared to chemotherapy. However, further research is needed to validate these findings.

## Data availability statement

The original contributions presented in the study are included in the article/supplementary material. Further inquiries can be directed to the corresponding author.

## Ethics statement

Patients included in this study underwent genetic counselling and signed written informed consent for their data and samples to be used for research purposes. Guiqian International General Hospital Ethics Committee had no objections against this study [project identification codes 2023 Audition No. (01)].

## Author contributions

Conceptualization, DD, GX and SZ. Methodology, DD. Formal analysis, WS. Investigation, DD, BJ, WW, QY, TY, MC, LZ and WS. Resources, GX and SZ. Data curation, DD and LZ. Writing—original draft preparation, DD. Writing—review and editing, DD, GX and SZ. Visualization, DD and WS. Supervision, GX and SZ. Project administration, DD. All authors contributed to the article and approved the submitted version.
