# Corrigendum: Correlation analysis of MRD positivity in patients with completely resected stage I-IIIA non-small cell lung cancer: a cohort study

**DOI:** 10.3389/fonc.2023.1275851

**Published:** 2023-08-25

**Authors:** Daling Dong, Shixin Zhang, Bin Jiang, Wei Wei, Chao Wang, Qian Yang, Tingzhi Yan, Min Chen, Liken Zheng, Weikang Shao, Gang Xiong

**Affiliations:** ^1^ Department of Cardiothoracic Surgery, Guiqian International Hospital, Guiyang, China; ^2^ Genecast Biotechnology Co., Ltd., Wuxi, China

**Keywords:** molecular residual disease, liquid biopsy, non-small cell lung cancer, adjuvant therapy, clinicopathological features, immune markers

In the published article, there was an error regarding the affiliation for the author Gang Xiong. Instead of Gang Xiong^1,2^*, it should be Gang Xiong^1^*.

In addition, there was an error in Supplementary Data Sheet 1.zip (Table 3). The Supplementary material contains sensitive information about the patient that has not been deleted, and some of the descriptions in the table have not been converted to English, which will affect the reader’s reading experience. The correct supplementary material statement appears below.

Table 3 Patient baseline information and MRD status

The authors apologize for these errors and state that these do not change the scientific conclusions of the article in any way. The original article has been updated.

