# Supplementary material for: Correlation analysis of MRD positivity in patients with completely resected stage I-IIIA non-small cell lung cancer: a cohort study
Source: Front Oncol. 2023 Jul 21;13:1222716. doi: 10.3389/fonc.2023.1222716 (PMC10401588; doi:10.3389/fonc.2023.1222716)
Supplement: Supplementary file 1 [file Table_1.docx]

**Supplemental Table 1 Patient clinical characteristics**

| Characteristic | All patients (N = 90) |
| --- | --- |
| Age (years)  median age  Sex (%)  male  female  Smoking history (%)  YES  NO  Pathological type (%)  LUAD  LUSC  TNM stage (%)  IA  IB  IIA  IIB  IIIA  Adjuvant therapy (%)  Chemotherapy alone  TP scheme  AP scheme  Targeted therapy  Gefitinib  Osimertinib  Chemotherapy + ICIs  TP regimen + tislelizumab | 55 (35-74)  39 (43.33%)  51 (56.67%)  32 (35.56%)  58 (64.44%)  78 (86.67%)  12 (13.33%)  41 (45.56%)  5 (5.56%)  1 (1.11%)  12 (13.33%)  31 (34.44%)  4 (4.44%)  20 (22.22%)  4 (4.44%)  8 (8.89%)  8 (8.89%) |

LUAD: lung Adenocarcinoma;LUSC:Lung Squamous Cells;TP scheme:nab-paclitaxel + platinum (cisplatin/carboplatin);AP:pemetrexed + platinum (cisplatin/carboplatin)
