# Supplementary material for: Correlation analysis of MRD positivity in patients with completely resected stage I-IIIA non-small cell lung cancer: a cohort study
Source: Front Oncol. 2023 Jul 21;13:1222716. doi: 10.3389/fonc.2023.1222716 (PMC10401588; doi:10.3389/fonc.2023.1222716)
Supplement: Supplementary file 2 [file Table_2.docx]

**Supplemental Table 2 Relationship between gene mutations and MRD status**

| gene mutation | MRD positive | MRD negative | *p* value |
| --- | --- | --- | --- |
| EGFR  TP53  BRAF  NTRK3  KRAS  ERBB2  ALK | 7  10  2  4  2  0  1 | 41  34  10  8  8  7  5 | 0.5731  0.0731  0.4817  0.1507  0.5897  0.2141  0.3112 |
